# Modeling succession of key resource-harvesting traits of mixotrophic plankton

**DOI:** 10.1038/ismej.2016.92

**Published:** 2016-08-02

**Authors:** Terje Berge, Subhendu Chakraborty, Per Juel Hansen, Ken H Andersen

**Affiliations:** 1 Department of Biology, VKR Centre for Ocean Life, Marine Biological Section, University of Copenhagen, Helsingør, Denmark; 2 National Institute of Aquatic Resources, VKR Centre for Ocean Life, Technical University of Denmark, Charlottenlund, Denmark

## Abstract

Unicellular eukaryotes make up the base of the ocean food web and exist as a continuum in trophic strategy from pure heterotrophy (phagotrophic zooplankton) to pure photoautotrophy (‘phytoplankton'), with a dominance of mixotrophic organisms combining both strategies. Here we formulate a trait-based model for mixotrophy with three key resource-harvesting traits: photosynthesis, phagotrophy and inorganic nutrient uptake, which predicts the trophic strategy of species throughout the seasonal cycle. Assuming that simple carbohydrates from photosynthesis fuel respiration, and feeding primarily provides building blocks for growth, the model reproduces the observed light-dependent ingestion rates and species-specific growth rates with and without prey from the laboratory. The combination of traits yielding the highest growth rate suggests high investments in photosynthesis, and inorganic nutrient uptake in the spring and increased phagotrophy during the summer, reflecting general seasonal succession patterns of temperate waters. Our trait-based model presents a simple and general approach for the inclusion of mixotrophy, succession and evolution in ecosystem models.

## Introduction

Photoautotrophic plankton combines photosynthesis with uptake of dissolved nutrients to convert CO_2_ and minerals into the biomass that fuels higher trophic levels in ocean food webs. The traditional view of planktonic ecosystems distinguishes between photoautotrophic ‘plants' and heterotrophic ‘animals' that acquire all essential resources through feeding on other organisms. However, in the microbial plankton, a mixotrophic strategy where photoautotrophy and ingestion of prey are combined in the same organism is widespread and often dominates large ecosystems (Burkholder *et al.*, 2008; Hartmann *et al.*, 2012; Flynn *et al.*, 2013; Hansen *et al.*, 2013).

Photoautotrophic plankton evolved from a strictly heterotrophic ancestor that acquired photosynthesis through endosymbiosis (Cavalier-Smith, 1982). Although the ecologically important diatoms lost their ancestral phagotrophy trait, most motile photoautotrophic plankton (or ‘phytoplankton') feed to different degrees (Flynn *et al.*, 2013). Driven by recent observations of the importance of mixotrophy in diverse aquatic ecosystems, a new plankton paradigm is emerging, where the base of aquatic food webs consists of organisms occupying the full spectrum from photoautotrophs to heterotrophs (Jones, 1994; Flynn *et al.*, 2013; Mitra *et al.*, 2014).

Despite several observations of mixotrophy since the 1980s, it has only recently been represented in plankton modeling studies (Thingstad *et al.*, 1996; Stickney *et al.*, 2000; Bruggeman, 2009; Flynn and Mitra, 2009; Ward *et al.*, 2011; Våge *et al.*, 2013; Mitra *et al.*, 2014). This is partly due to the reliance on functional-group type of modeling paradigms, where organisms are pre-described as ‘phytoplankton' and ‘zooplankton'. Representing mixotrophy in such models leads to increased complexity and computational costs. However, by disposing of functional groups and species altogether, and focusing on the distribution of continuous trait values, trait-based approaches have the potential to represent the full spectrum of trophic strategies and partly overcome this complexity problem (Norberg *et al.*, 2001; Bruggeman, 2009; Andersen *et al.*, 2015).

Our aim is to understand which environmental conditions favor specific key resource-harvesting traits or trophic strategy of mixotrophic plankton. To this end, we model a general organism where the trophic strategy is not prescribed *a priori*, but is an emergent property. The emergent trophic strategy is defined by the relative investment in three traits: photosynthesis, inorganic nutrient uptake and phagotrophy.

A large proportion of mixotrophs, especially the constitutive mixotrophs that synthesize their own chloroplasts (Mitra *et al.*, 2016), show an obligate energy dependence on sunlight for both phagotrophy and growth (Hansen, 2011). We assume that small carbohydrates from photosynthesis represent the only source of carbon for respiration in these mixotrophs. Phagotrophy primarily provides nutrients and carbon for synthesis, whereas photosynthesis provides carbon for both synthesis and respiration. We consider flows of both carbon and nitrogen, and three resource-harvesting traits; inorganic nutrient uptake, phagotrophy and photosynthesis. In this way we go beyond earlier trait-based approaches that only considered either one essential nutrient or carbon (Thingstad *et al.*, 1996; Bruggeman, 2009; Våge *et al.*, 2013), and thus had no ability to reproduce synergistic effects of mixotrophs, gained through labor sharing on alternative nutrient and carbon sources (Mitra *et al.*, 2014).

The development and parameterization of our model was guided by laboratory observations on two mixotrophic dinoflagellates of the genus *Karlodinium: K. armiger* and *K. veneficum*. The species were chosen based on the large amount of available laboratory data and knowledge on trait variation in this important group of mixotrophs (Berge *et al.*, 2008a, b; Li *et al.*, 1999, 2000; Adolf *et al.*, 2006; Calbet *et al.*, 2011; Berge and Hansen, 2016). The two species have approximately the same investments in photosynthesis (Adolf *et al.*, 2006; Berge and Hansen, 2016). *K. armiger* seems to invest very little in the uptake of inorganic nutrients, but relies heavily on phagotrophy (Berge *et al.*, 2008b, 2012). *K. veneficum* has a lower affinity and maximum uptake rate for food, but higher capacity to take up inorganic nutrients and can achieve fairly high growth rates without food in standard nitrate-enriched laboratory media (Li *et al.*, 1999; Adolf *et al.*, 2006). In the model, we represent these species-specific differences in trophic strategy as differences in trait values representing relative investment in photosynthesis, phagotrophy and inorganic nutrient uptake.

Even though we use two species of *Karlodinium* as a case study, our approach represents a general system as the fundamental parameters quantifying the trade-offs are species-independent constants. We apply the model to examine which trait combination results in the highest population growth rates in a given environment and during a seasonal succession. The results support a hypothesis that the trophic strategy of mixotrophic populations change from higher investment in photosynthesis and inorganic nutrient uptake in the spring (photoautotrophy) to higher investments in phagotrophy during the summer conditions.

## Model description

The model represents a mixotrophic cell that can acquire (1) inorganic carbon from photosynthesis, (2) inorganic nutrients by the activity of membrane-bound uptake sites, and (3) organic carbon and nutrients by phagocytosis. The cells' ability to take up these resources, that is, the affinities and maximum uptake rates, are determined by investments (*φ*) in organelles and enzymes associated with each function, and described by the three key resource-harvesting traits: photosynthesis *φ*_L,_ (for example, pigments and enzymes for carbon fixation), phagocytosis *φ*_F_ (for example, the microtubule-supported peduncle, membrane material for food vacuole formation and digestive enzymes) and enzymes for the uptake of inorganic nutrients *φ*_N_ (for example, enzymes for reduction of nitrate to ammonium and transporter enzymes). The model structure follows Bruggeman and Kooijman (2007) and Bruggeman (2009). There are some differences to the model by Bruggeman (2009): here, we generalize to three resource-harvesting traits, compared with only two traits of Bruggemann (2009) (photosynthesis and phagotrophy). Further, he did not distinguish between carbon and essential nutrient flow explicitly, and consequently nutrients (for example, nitrogen or phosphorous) were respired (lost) in his model. We avoid this by keeping explicit track of both carbon and one essential nutrient. We consider nitrogen as the essential element in addition to carbon, and assume only one source of dissolved inorganic nitrogen.

**Figure 1:**
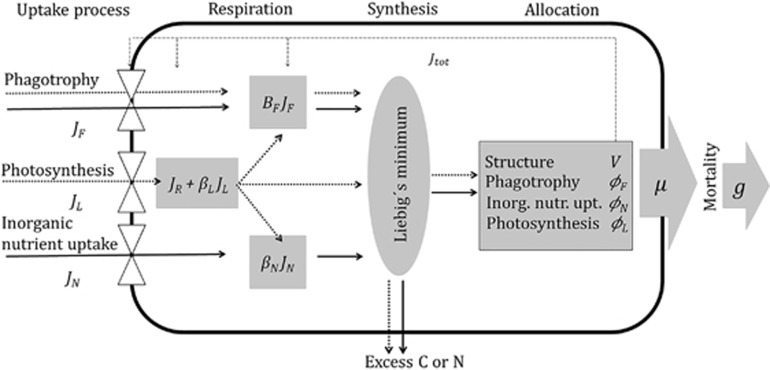
Schematic representation of the model showing how fluxes of carbon (dotted) and nitrogen (solid) are lost through respiration (small gray rectangles), and combined (gray ellipse) to traits and structure (large gray rectangle). Traits, structure and food are assumed to have the same carbon/nitrogen ratio. White triangle symbols represent the functional responses for the uptake mechanisms. Phagotrophy and inorganic nutrient uptake activity depends on energy from sunlight. *J*_R_ includes the basal respiratory costs of synthesis and maintenance of all traits and structure, and is paid before the uptake of food and inorganic nutrients. Uptake and mobilization for synthesis (or fluxes *J*_*i*_'s) need to pay a respiratory cost represented by a fraction of the gross flux of each pathway. The ellipse represents synthesis of biomass from the available carbon and nutrients following Liebig's law of the minimum and constrained by the Redfield ratio (gC/gN=5.7). In our steady state consideration, a proportion of the assimilated carbon or nutrients are assumed lost as excess resources. Long dashed thin arrows illustrate how resource allocation into traits and structure regulates uptake affinities and respiratory costs.

Fluxes of carbon and nitrogen are described by symbol *J*_*i*_ (mass flows *i* being food (F), carbon from photosynthesis (L) or nutrients (N) (see Table 1 for central symbols and parameters), which are combined to synthesize the new biomass; Figure 1). Respiratory costs of fluxes, *β*_*i*_*J*_*i*_ include costs of both uptake and mobilization for synthesis through each pathway. Biomass synthesis rate *J*_tot_ is constrained by the stoichiometric balance between carbon and nitrogen. We assume a constant C:N ratio of both the mixotroph and the food. Moreover, we assume that traits and structure have similar stoichiometry and basal maintenance costs. Finally, we assume that carbon for respiration is acquired from photosynthesis only. The final assumption is needed to represent the observed light-dependent feeding common among constitutive mixotrophs.

### Investment in resource-harvesting traits

The biomass of the cell is divided into four pools: cell structure *V* (units of gC), photosynthetic machinery *φ*_L_*V*, machinery for inorganic nutrient uptake *φ*_N_*V* and machinery for phagotrophy *φ*_F_*V*. The three traits that we consider here, *φ*_L_, *φ*_N_ and *φ*_F_, are all dimensionless quantities, representing the investment into organelles as a fraction of the structural mass of the cell. The total mass of the cell is therefore (1+*φ*_L_+*φ*_N_+*φ*_F_)*V*. Each investment provides a benefit in terms of a higher affinity toward the resource (light/carbon, inorganic nutrients or food) and higher maximum uptake rate. The affinity is an increasing but saturating function of the investment:


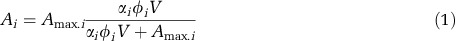


**Figure 2:**
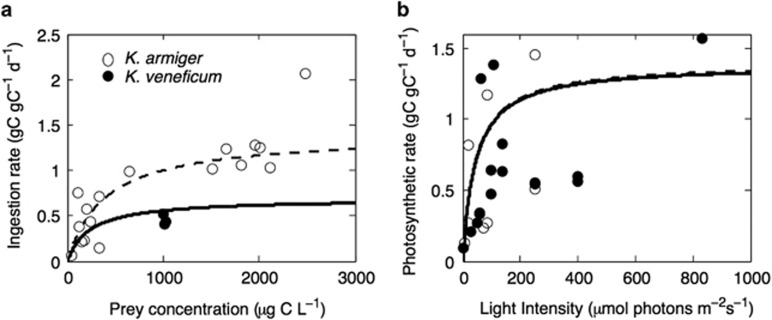
Functional responses of feeding (**a**) and photosynthesis (**b**). Observed data points and modeled lines for *K*. *veneficum* (solid) and *K*. *armiger* (dashed and open symbols). The medium was supplied with 838 μM inorganic nitrogen (**a** and **b**), prey concentrations were >800 μg C L^−1^ (**a**) and irradiance was above 180 μmol photons m^−2^ s^−1^ (**a** and **b**). Points are means of three replicate cultures. Data from Li *et al.*, 1999; Adolf *et al.*, 2006; Berge *et al.*, 2008b; Berge and Hansen, 2016.

*A*_max.i_ is the maximum affinity and *α*_*i*_ is the amount of affinity gained per investment *φ*_*i*_. The saturation of the affinity as a function of the investment represents the packaging effect for investment in light harvesting (Morel and Bricaud, 1981) and diffusion limitation for inorganic nutrient uptake. Besides leading to a higher affinity *A*_*i*_, investments also result in a higher maximum uptake rate *J*_max.i_:


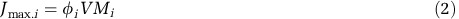


where *M_i_* is the maximum uptake rate per investment. Maximum uptake rates are not, as the affinities, limited by physical constraints leading to diminishing returns as in Equation (1). Rather, maximum uptake rates are limited by processing machinery (for example, chloroplasts, digestive and transporter enzymes), whose capacity we assume scales linearly with investments *φ_i_*(Figure 2).

### Costs

The investments in the traits have respiratory costs *J*_R_ in terms of maintenance of the organelles proportional to the investments *φ*_*i*_ (gC per day):


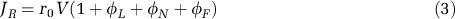


and of course the resource costs of synthesis *φ*_*i*_*V* (gC).

### Resource uptakes and growth rates

The potential uptake 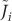 of resource *X*_*i*_ is governed by a standard saturating functional response:


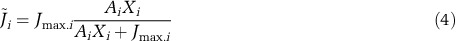


All fluxes have a respiratory cost *β*_*i*_ proportional to the actual flux *J*_*i*_. These costs include respiratory costs of uptake and mobilization for synthesis through the specific trait or pathway. Therefore, the potential uptake can only be realized if there is sufficient carbon available from photosynthesis to fuel the respiratory costs of inorganic nutrient and food uptakes. In periods of light limitation, carbon taken up by photosynthesis may be insufficient to support the energy needed to drive the potential uptake of inorganic nutrients or food. Those uptakes are therefore reduced by a light-energy-dependent factor *ρ* taking values between 0 and 1:


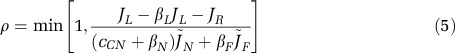


This relation assumes that maintenance metabolism is supported before remaining fixed carbon is respired to fuel inorganic nutrient and food uptake. The actual uptakes of nutrients *J*_N_ and food *J*_F_ are:


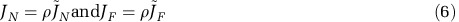


For photosynthesis, the actual uptake is the same as the potential uptake 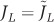.

Carbon and nitrogen are combined to synthesize new structure and investments in traits. This combination should respect homeostasis (that is, constant C/N ratio). The combination of carbon and nitrogen is performed following Liebig's law of the minima:


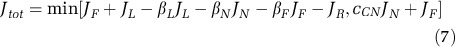


Note that this implies that either some excess carbon or nutrients are excreted and lost from the cell. The specific rate of synthesis *μ* then becomes


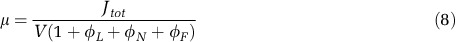


and the final growth rate *g* of the cells (d^−1^):


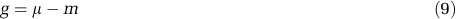


where *m* is the natural mortality rate.

### Parameters and trait values

Parameters for food uptake and photosynthesis were based on laboratory measurements on *K. veneficum* and *K. armiger* (Figure 2). Data for potential ingestion rate as a function of prey concentration were taken from experiments that used the same method for determining steady state ingestion rates in high light intensities (Li *et al.*, 1999; Adolf *et al.*, 2006; Berge *et al.*, 2008b; Berge and Hansen, 2016). This method is based on the disappearance of prey determined from cell counts in mixed cultures, and may overestimate the ingestion rate due to incomplete or ‘sloppy' feeding. This is especially the case for species like *Karlodinium* that use feeding tubes to pierce the prey before sucking in the cytoplasm (Berge *et al.*, 2008a), and prey membrane material containing various cell-fractions are sometimes left behind. We accounted for this by converting reported ingestion rates with a feeding efficiency factor of 0.7 for both species. Data for photosynthetic rates were derived from measurements using ^14^C light and dark incubations (Li *et al.*, 1999; Adolf *et al.*, 2006; Berge and Hansen, 2016). To better compare the laboratory data between the two species, which were obtained under different temperatures (15–20 °C), the data for *K. veneficum* (Li *et al.*, 1999; Adolf *et al.*, 2006) were normalized to 15 °C using a Q10 of 2.5.

We determine the central parameters (Table 1) of the trade-offs related to the benefits (the affinity, Equation (1), the maximum uptake rate, Equation (2), and the respiratory costs, Equation (3)) using the measurements of functional responses and growth rates as a function of light and food for the two *Karlodinium* species (Li *et al.*, 1999; Adolf *et al.*, 2006; Berge *et al*, 2008b; Berge and Hansen, 2016). We assume that the differences between the two species are represented only by differences in the three species-specific trait values *φ*_L_, *φ*_F_ and *φ*_N_ (Table 1). Guided by the arguments of Raven (1984, 1997), we assume that the costs of synthesizing the photosynthetic machinery may account for up to 50%, whereas the phagotrophic machinery accounts for <10% of the cell's total energy, carbon and nutrients. Moreover, we assumed that the total investments in the three resource-harvesting traits cannot exceed the investment in structure. To find the maximum affinities gained per investment (*A*_max.i_), we assume that affinity saturates at an investment of 0.9*V* for photosynthesis (=0.9 corresponding to 45% of total cell carbon and nutrient resources), around 0.4*V* for phagotrophy and 0.2*V* for inorganic nutrient uptake. This information, together with the observed half-saturation coefficients for photosynthesis and food uptake make it possible to estimate the maximum affinities (*A*_max.L_, *A*_max.N_ and *A*_max.F_) and the affinity gained per investment for photosynthesis and food uptake (*α*_L_, *α*_N_ and *α*_F_) (Appendix A); (Figure 2).

We assume that the structural size is the same between the two species, although laboratory reports suggest that *K. armiger* is slightly larger. Investment in the photosynthetic machinery, *φ*_L_, was assumed equal for the two species at 0.45 (Figure 2b). Investments in phagotrophy *φ*_F_ were assumed approximately two times higher in *K. armiger* than in *K. veneficum* (Figure 2a), and were set to 0.16 and 0.08, respectively (Li *et al.*, 1999; Adolf *et al.*, 2006; Berge *et al.*, 2008b; Berge and Hansen, 2016). *K. armiger* (*φ*_N_=0) has a very low investment in inorganic nutrient uptake, compared with *K. veneficum* (*φ*_N_=0.1), as it cannot grow in nitrate-enriched laboratory media (that is, L and F/2) without food (Berge *et al.*, 2008a, b; Berge and Hansen, 2016).

On the basis of growth efficiencies of strictly photoautotrophic and phagotrophic specialists (Straile, 1997; Falkowski and Raven, 2013), we assume the metabolic cost parameters of uptake and mobilization for synthesis through the photosynthetic pathway to be lower (*β*_L_=0.35) than through the phagotrophic pathway (*β*_F_=0.5). The respiratory cost of inorganic nutrient uptake and mobilization for growth (*β*_N_) was assumed to be 3 gC gN^−1^, which is similar to that of nitrate acquisition (see for example, Flynn and Mitra, 2009). The basal respiration rates of maintenance were assumed to be similar and *r*_0_=0.05  d^−1^ (see for example, Flynn and Mitra, 2009) for both structure and the three investments.

The model is used first to describe uptake and growth rates of the two *Karlodinium* species. In this part, the set of traits take fixed values for each species. Next, we analyze which combinations of trait values {*φ*_L_,*φ*_F_,*φ*_N_} are optimal, that is, that maximize population growth rate under various environmental conditions. For the maximization, we enforce that the total investment cannot exceed the structural mass: ∑*φ*_*i*_⩽1.

## Results

**Figure 3:**
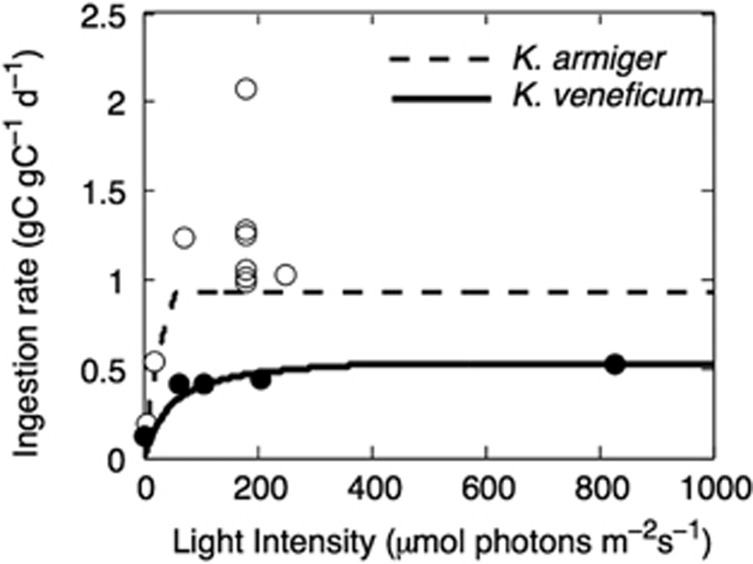
Light-dependent steady state ingestion rates as a function of irradiance in *K*. *armiger* (open symbols) and *K*. *veneficum* (closed symbols). Points are means of the three replicate laboratory cultures fed saturating prey concentrations (>1000 μg C L^−1^). Data from Li *et al.*, 1999; Berge and Hansen, 2016.

**Figure 4:**
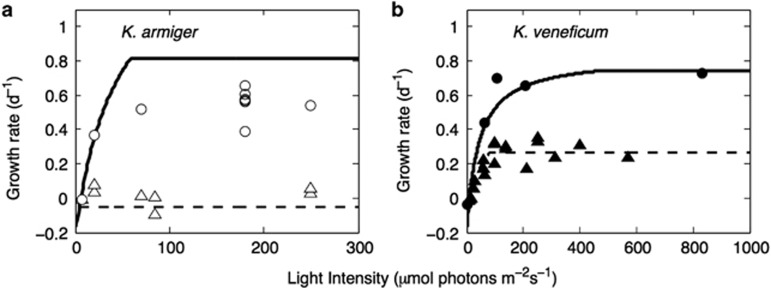
Growth rates in monocultures (dashed) and in fed cultures (solid) for (**a**) *K. armiger* and (**b**) *K. veneficum* as a function of light intensity in food and inorganic nitrogen-saturated cultures. Observed growth rates in monoculture (triangles) and (circles) are means of three replicates. Data from Li *et al.*, 1999; Adolf *et al.*, 2006; Berge and Hansen, 2016.

With fixed trait values *φ*_L_, *φ*_F_ and *φ*_N_, the model reproduces fundamental observations of the two model species from the laboratory, including ingestion (Figure 3) and growth rates (Figures 4a and b) as functions of irradiance in fed and unfed cultures (grown in high inorganic nitrogen media). Ingestion rates in high food concentrations are close to zero under low irradiance, but increases with irradiance (Figure 3). In effect, growth rates are negative under very low irradiations, even with plenty of food and inorganic nutrients.

Without food, growth increases as functions of irradiance only in *K. veneficum*, whereas growth is not possible in *K. armiger*, due to the low investment in inorganic nutrient uptake (*φ*_N_)); (Figures 4a and b), and the lack of prey and thus nutrient limitation. The modeled mixotrophic growth rates of *K. armiger* under high food, high inorganic nutrients and light levels were slightly higher than the observed growth rates (Figure 4a), whereas the growth rates under low light intensity were slightly underestimated.

**Figure 5:**
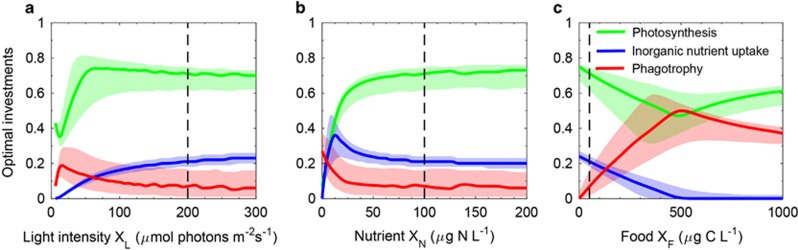
The combination of traits yielding the highest growth rate as functions of light (**a**), inorganic nutrients (**b**) and prey concentration (**c**) in constant environments. Dashed lines indicate the base value of resource, e.g., in (**a**), dashed line represents the level of inorganic nutrients and food in panels **b** and **c**. Shaded areas represent the range of trait values giving growth rates within 95% of optimal growth rates, and suggest environmental states where trait values are likely to vary (e.g., genotypic plasticity or intraspecific variation).

The trait values {*φ*_L_,*φ*_F_,*φ*_N_} that result in the highest population growth rate depend on the environment (Figure 5). Optimal investment in light harvesting is generally high (*φ*_L_, 0.45–0.90), but decreases with irradiance in an environment with limited food and high levels of inorganic nutrients (Figure 5a). In high light and low food levels, optimal investment in inorganic nutrient uptake increases with nutrient concentration, but reach a maximum at low levels. It further decreases at high nutrient levels (Figure 5b). Under high light conditions, optimal investments in phagotrophy increase with prey concentration and reach a maximum of 0.5 at intermediate prey concentrations. At higher prey concentrations, investment in phagotrophy further declines (Figure 5c). A wide range of combinations of trait values yield growth rates within 95% of the optimal growth rate (shaded areas in Figure 5).

**Figure 6:**
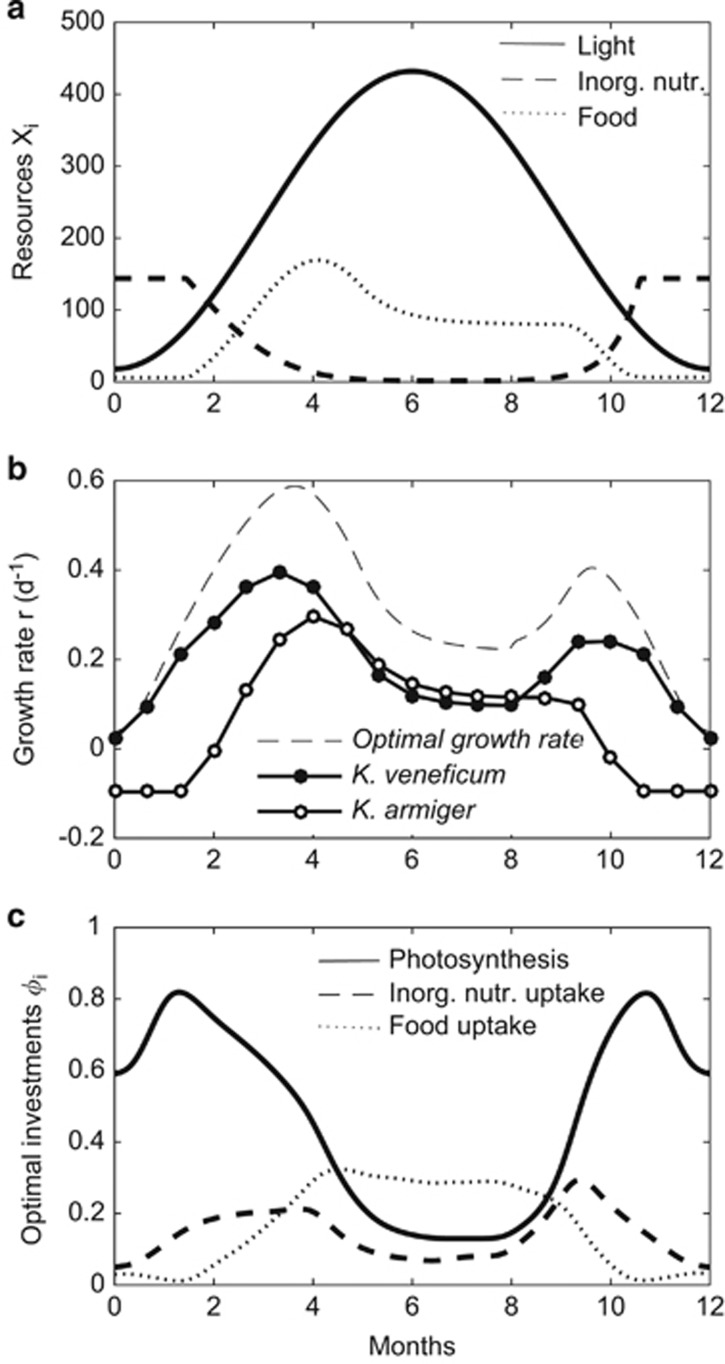
Seasonal succession in surface waters of a high-latitude plankton community. (**a**) Assumed seasonal variation in light (solid), inorganic nutrients (dashed) and prey (dotted) in the idealized coastal setting. *X*_*i*_ refers to either μmol photons m^−2^ s^−1^,  μg N L^−1^ and μg C L^−1^. (**b**) Modeled growth rates of *K*. *armiger* (white), *K*. *veneficum* (black) and of an optimally investing species (dashed). (**c**) The combinations of traits yielding the highest growth rate (optimal growth rate in **b**) throughout the season illustrate the succession of resource-uptake traits, i.e., the trophic strategy.

We calculated growth rates of *K. armiger* and *K. veneficum* in a constructed temperate seasonal cycle, from new production in the spring (high inorganic nutrients) to recycled production during summer (moderate food levels); (Figure 6a). The model shows that *K. veneficum* will outcompete *K. armiger* in the beginning and end of the season, whereas *K. armiger* attain higher competitive abilities later in the summer, when inorganic nutrients are depleted and organic food levels are moderately high (Figure 6b).

The growth rate of an optimally investing species is positive throughout the season. During spring and at the end of the growing season, the optimal trait combination is a low investment in phagotrophy, and high investment in inorganic nutrient uptake and light harvesting (Figure 6c). Investment in light harvesting is high at low irradiance during the winter, but declines under high summer irradiances. Optimal investment in phagotrophy shows highest levels during the summer and declining to very low during winter.

**Figure 7:**
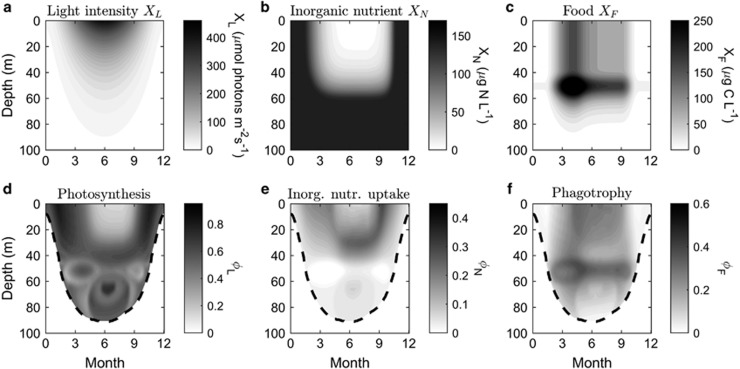
Seasonal succession of trophic strategy in a constructed idealized temperate stratified plankton system with imposed variations of (**a**) light, (**b**) inorganic nutrients and (**c**) food. (**d–f**): combinations of resource-uptake traits giving the maximum population growth rate. Growth rates are negative in the white area below the dashed lines (the light compensation depth).

To further understand the succession of trophic strategies in plankton communities, we modeled the optimal investments in resource-uptake traits as a function of depth throughout the season. We constructed a seasonal cycle in an idealized plankton system with a stable pycnocline at 50 m depth, and a light compensation point at 80 m during the summer and a mixed water column during the winter. Surface irradiance followed the seasonal pattern and penetrated water from a few meters in the winter to the depth of 80 m during the summer (Figure 7a). We assumed a constant light extinction throughout the season. We let the concentration of inorganic nutrients quickly to become depleted after the spring bloom in the photic zone. Inorganic nutrients increased with depth below the pycnocline and reached highest level where light was still present. In the late autumn, when the light decreased, inorganic nutrients returned to high winter and spring levels (Figure 7b). A cryptophyte prey population formed a subsurface maximum throughout the summer stratification, with typical summer concentrations above the pycnocline (Figure 7c). This system represents a typical temperate seasonal succession in inorganic nutrients (dissolved inorganic nitrogen), food and light levels in a coastal setting (for example, Irigoien *et al.*, 2005).

The general relationship of decreased optimal investment with increased resources (Figure 5) was reflected in the optimal trait combinations in the constructed water column (Figures 7d–f). As light penetrates deeper during the course of the season, a high investment in photosynthesis is optimal at low light intensities at larger depths. A lower investment in phagotrophy is optimal in the subsurface prey population, whereas a high investment in phagotrophy and low investment in inorganic nutrient uptake (that is, similar to *K. armiger*) is optimal in surface waters during the summer. Investment in both inorganic nutrient uptake and phagotrophy, similar to *K. veneficum*, is optimal at larger depths during the summer and in the surface during the spring and autumn (Figures 7d and f). Allocating resources toward phagotrophy at the expense of inorganic nutrient uptake is optimal during the summer, when optimal investments in photosynthesis and inorganic nutrient uptake are low (Figures 7d and f).

## Discussion

We have developed a general trait-based model of mixotrophs that is able to represent a spectrum of trophic strategies. The model builds on existing simple trait-based models of mixotrophy (Bruggeman, 2009; Ward *et al.*, 2011; Våge *et al.*, 2013), but goes beyond these by introducing three variable traits and by explicitly resolving the flows of carbon and one essential nutrient (here, nitrogen).

The model reproduced the observed obligate light dependence of phagotrophy in *Karlodinium* spp. (Figure 3), and growth rates under different light and food scenarios also reproduced well with reported data from laboratory observations for the two model species (Figure 4). This shows that a reliance of photosynthates for respiration may explain the typical obligate dependence of light in constitutive mixotrophs.

### Model simplifications

Despite the simplicity of the model, it was able to recreate the main differences in the functional responses between the two *Karlodinium* species under steady state conditions. Therefore, most of the variability between the two species can be captured by just two traits representing their difference in the investments in inorganic nutrient uptake and phagotrophy. The fits were not perfect though. Our model generally produced slightly lower growth rates under resource limitation and slightly higher growth rates under resource saturation compared with laboratory observations (Figures 4a and b). These differences likely originate because we keep the trait values constant. In reality, trait values would be able to change toward the optimum within the limits of phenotypic plasticity, in response to varying light, inorganic nutrients and prey levels (Li *et al.*, 2000). For example, photosynthetic plankton short-term acclimate to low irradiance by producing more pigment, that is, investing more in phototrophy in the matter of hours to days. For example, in *K. veneficum* and *K. armiger*, cellular chlorophyll levels increase by a factor of three to five times in light-limited compared with light-saturated conditions (Li *et al.*, 1999; Adolf *et al.*, 2006; Berge and Hansen, 2016). Nutrient limitation and food concentration or ingestion rate have also been found to affect chlorophyll levels in other mixotrophic dinoflagellates; (Skovgaard, 1996; Hansen, 2011). In general, such changes can be represented by letting the trait values vary and reflect adaptation to the changing environmental conditions.

Despite observations of substantial variation of stoichiometric ratios around Redfield ratios, for example, 20-fold for the N:P ratio (Rhee and Gotham, 1980; Klausmeier *et al.*, 2004), we have used constant stoichiometric ratios for simplicity. Non-Redfield ratios are products of several processes: non-Redfield costs of investments in traits (Klausmeier *et al.*, 2004) and reserves, or non-Redfield ratios of prey organisms. Including such variable stoichiometry between structure, traits and prey in our model would probably better reflect the benefits of feeding in mixotrophs like *Karlodinium* spp. For example, Li *et al.* (2000) measured two times higher C:P ratios in *K. veneficum* than in the cryptophyte prey *Storeatula major*, which would double the modeled growth rate of the mixotroph. Moreover, nutrient limitation also affects stoichiometry. In an environment with limited concentration of food, nitrogen starved *K. armiger* may have C:N ratios much higher than its prey. This suggests a larger effect of feeding on the growth in nutrient-limited cells than reflected by our general model. The model may be extended to account for further physiological details, for example, additional essential elemental nutrients, non-Redfield ratios, ammonium–nitrate interactions, reserves and feedback responses (Flynn and Mitra, 2009) by including more traits. However, this will be at the cost of computational simplicity, a prerequisite for the inclusion in larger models, for example, food-web and global circulation models using current computer abilities.

### Optimal trait values and seasonal succession

The trait-based approach let us generalize beyond the level of species, by loosening the assumption of constant trait values from our comparison between *K. armiger* and *K. veneficum*. Depending on the timescales under consideration, the model output has the potential to address species plasticity (acclimation), plankton succession, evolution of general populations and speciation. Thus, on short timescales, photoacclimation emerges from the model output as a high investment in photosynthesis at low irradiance (Figure 5a and 7). At longer timescales, our model predicts a seasonal succession from photoautotrophy to phagotrophy as the dominating trophic strategy (Figure 6 and 7).

We used optimization to generalize beyond the two model species. A fundamental requirement of optimization and selection is the presence of trait variation. We found that a wide range of combinations of trait values will yield growth rates almost on the same level as the optimal level (Figure 5). This means that selection for a specific type of organism is weak, and therefore, a large diversity of organisms can be expected to coexist. In the case of our model species, both laboratory and field evidence show that natural populations contain large amounts of intraspecific trait variation in the key resource-harvesting traits considered in our model. Even within the same population of a single species, strain variation in mixotrophic configuration is very large (Bachvaroff *et al.*, 2009; Berge, 2011; Calbet *et al.*, 2011). Thus, there is plenty of diversity in trophic strategy within species for seasonal succession.

Our modeled optimal trait configuration throughout the season showed that *K. veneficum* would outcompete *K. armiger* in the spring, whereas the opposite is the case during the summer, when inorganic nutrients are depleted and food levels are moderate. The same pattern was observed from the optimal combination of traits. The optimal trophic strategy of mixotrophic populations changed from a high investment in photosynthesis and inorganic nutrient uptake in the spring (photoautotrophy) to high investment in phagotrophy during summer conditions. This qualitatively reflects general aspects of community plankton succession in temperate seasonal cycles, which typically involves an initial spring bloom of photoautotrophic diatoms with high investments in photosynthesis and inorganic nutrient uptake (Litchman *et al.*, 2007), followed by a community of heterotrophic and mixotrophic flagellates during the ‘clear-water' summer period. This pattern may also reflect general spatial trends in trophic strategy, such as across frontal upwelling and estuarine areas.

### Obligate dependence on sunlight

The precise mechanism behind the light dependency of several constitutive mixotrophs is unknown (Hansen, 2011). However, in *K. armiger*, prey capture and ingestion takes place in the dark, but digestion and/or assimilation stop, and the dinoflagellates will not survive (Berge *et al.*, 2008a). In *K. veneficum*, laboratory experiments report inorganic nitrogen and prey uptake rates close to zero in the dark (Paasche *et al.*, 1984; Li *et al.*, 1999), suggesting a lack of energy. Either light provides essential energy through unknown pathways (for example, rhodopsins) or ‘photoheterotrophy' is involved (that is, light harvesting without carbon fixation, but generation of reducing energy); (Wilken *et al.*, 2014), or that mitochondria prefer carbohydrates from photosynthesis for respiration (Putt, 1990).

Our model assumed that the ability to break down and use food for respiration is lacking. This is a controversial assumption, given that mixotrophs evolved from heterotrophic ancestors where this ability is obviously present. A potential loss implies a relatively higher cost of breaking down organic matter to simple molecules for respiration compared with using simple sugars from photosynthesis (Putt, 1990). A potential loss of ability to use prey-derived carbon for growth may also imply very high benefits of respiring low-molecular carbohydrates in the light, where these organisms thrive. Although the majority of marine constitutive mixotrophs are dependent on sunlight, a few species can survive exclusively heterotrophic in the dark (Skovgaard *et al.*, 2000; Hansen, 2011; Mitra *et al.*, 2016). Recently, Calbet *et al.* (2011) reported survival for several months in the dark of a single strain of *K. veneficum*. Our model may be extended to represent the full spectrum from pure phototrophy to pure heterotrophy, by including the phagotrophic respiratory pathway. It is to be expected, then, that mixotrophic organisms will outcompete specialist phagotrophs by surviving at lower food concentrations in the photic zone. Specialist phagotrophs would be able to outcompete mixotrophs under low light conditions with enough food. Such a model would, however, not adequately represent the majority of naturally observed mixotrophic strategies (Stoecker, 1998; Mitra *et al.*, 2016), and be unable to represent obligate light-dependent feeding.

### Model application

The simplicity of the model allows it to be deployed easily to predict the dominating mixotrophic strategy under given conditions or used as a basis for dynamic simulations of trait dynamics in time and space (Bruggeman, 2009). Our model organisms, *Karlodinium* spp., use toxins to immobilize prey before feeding and are well known as some of the most problematic species for aquaculture in coastal areas worldwide (Sheng *et al.*, 2010). The toxins are strong enough to allow ingestion of metazoan grazers, for example, copepods, and *Karlodinium* blooms may potentially turn the food web upside down (Berge *et al.*, 2012). As the model predicts when and where we might expect *Karlodinium spp*. populations to invest heavily in phagotrophy, it may help us understand critical periods for aquaculture in areas where these species exist.

Our study is purely bottom-up focused (that is, resource harvest), without considering trophic interactions and mortality losses such as predation (top-down effects). In a food-web context, strong trade-offs may exist between risk of being eaten and investment in phagotrophy, or the risk of virus attacks and investment in inorganic nutrient uptake (Våge *et al.*, 2013). Unicellular organisms are characterized by several traits affecting mortality, for example, feeding mode (ambush vs cruising; Kiørboe, 2011), motility, toxin production or defense against viruses. Thus, the implementation in a food-web model needs identification of additional ‘key traits' involved in biotic interactions. Traits and trade-offs centered around mortality are more difficult to quantify experimentally than resource-harvesting traits.

## Conclusion

We have demonstrated how trait-based modeling techniques can succinctly describe the main differences between mixotrophic plankton by just a few well-chosen traits. Here, our focus has been on traits related to resource uptake. A future challenge to this approach will be to include other traits, for example, cell size, as well as traits not directly related to resource harvesting, for example, defense traits. Although the trait-based approach will not replace current species- or functional-group-based approaches, it is useful for understanding the broad-scale patterns in global or seasonal changes in plankton communities.

## Figures and Tables

**Table 1:** Central symbols and general parameters

*Symbol*	*Description*	*Value and unit*
*Central symbols*		
*X*_L_	Light flux in the environment	μmol photons m^−2^ s^−1^
*X*_N_	Concentration of inorganic nutrients in the environment	μg N L^−1^
*X*_F_	Concentration of food in the environment	μg C L^−^^1^
*A*_L_	Affinity for light	μg C (μmol photons m^−2^ s^−1^)^−1^
*A*_N_	Affinity for inorganic nutrients	L d^−1^
*A*_F_	Affinity for food	L d^−1^
*J*_*i*_	Flux of assimilated substance or respiration	μg C L^−1^ or μg N L^−1^
*Biomass and traits*		
*φ*_*i*_	Traits	Variable (μgC μgC^−1^)
*V*	Structural mass	6.50 × 10^−5^ μg C
*Functional responses*		
*A*_max.L_	Maximum affinity for light	4.07 × 10^− 6^ μg C (μmol photons m^−2^ s^−1^)^−1^
*A*_max.N_	Maximum affinity for inorg. nutr.	1 × 10^−6^ L d^−1^
*A*_max.F_	Maximum affinity for food	4.65 × 10^−7^ L d^−1^
*α*_L_	Affinity per investment in photosynthesis	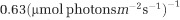
*α*_N_	Affinity per investment in inorg. nutr. upt	0.30 L d^−1^ (μg C)^−1^
*α*_F_	Affinity per investment in phagotrophy	0.16 L d^−1^ (μg C)^−1^
*M*_L_	Maximum uptake rate per investment in photosynthesis	5.01 L d^−1^
*M*_N_	Maximum uptake rate per investment in inorganic nutrient uptake	1.00 (μg N μg C^−1^) d^−1^
*M*_F_	Maximum uptake rate per investment in phagotrophy	14.10 d^−1^
*Costs*		
*β*_L_	Cost of photosynthesis	0.35 μg C μg C^−1^
*β*_N_	Cost of inorg. nutr. upt. and synthesis	3.00 μg C μg N^−1^
*β*_F_	Cost of food uptake and synthesis	0.50 μg C μg C^−1^
*r*_0_	Basal respiration rate	0.05 d^−1^
*Synthesis and growth*		
*m*	Mortality	0.12 d^−1^
*c*_CN_	C/N ratio in food and in the cell	5.68 μg C μg N^−1^
*Trait values*		
*φ*_L_	Investment into light harvesting	0.45 *K. armiger*
		0.45 *K. veneficum*
*φ*_F_	Investment into phagotrophy	0.16 *K. armiger*
		0.08 *K. veneficum*
*φ*_N_	Investment into inorganic nutrient uptake	0.00 *K. armiger*
		0.10 *K. veneficum*

Index *i* refers to light (L) measured in units of μmol photons m^−2^ s^−1^, inorganic nutrients (inorg. nutr.) (N) in units of μg N L^−1^and food (F) in units of μg C L^−1^. Upt. refers to uptake. All parameters are derived in the section ‘Parameters and trait values' and in the Appendix.

## Appendix A

### Appendix A Functional response and trait values

Figure A: Illustration of how the functional response depends on the resource concentration for different values of the investment. The maximum response increases linearly with the investment, whereas the affinity (slope at origin) only increases up to a maximum indicated by the dotted line. Parameters used in the example are for photosynthesis (Table 1).

### Calibration of parameter values for affinities and functional response

The functional response contains three species-independent fundamental parameters and the trait value *φ*_*i*_. Two parameters concern the affinity (*A*_max.i_ and *α*_*i*_), and one the maximum uptake rate (*M*_*i*_). We assume that the trait value is known and need to determine the three others. The information comes from measurements of the functional response, which provides two parameters: maximum uptake rate *J*_max.i_ and half-saturation constant *K*_*i*_. The constant involved in maximum uptake rate follows directly from Equation (2):

Determining the two parameters concerning the affinity requires one more piece of information concerning the level of investment,*φ*_sat.i_, that leads to saturation of the affinity (Equation (1)), that is, at which investment is the pay-off of investment significantly diminished. We assume that saturation happens when the affinity is around 90% of maximum affinity, that is, when *A*_*i*_=*c*_sat_*A*_max.i_, with *c*_sat_=0.9. The half-saturation constant is given as the ratio between the affinity and the maximum uptake rate, *K*=*J*_max.i_/*A*_*i*_. Taken together, this information provides expressions for the two affinity parameters:

For light affinities, we assume *φ*_sat.L_=0.9 and get *J*_max.L_≈1.4*V* (1+*φ*_L_+*φ*_N_+*φ*_F_) μg_C_ per day and *K*≈40 μmol photons m^−2 ^s^−1^ from Figure 2b. This gives *M*_L_≈5.012, *α*_L_≈ 0.6265  and .

For food uptake affinities, we assume *φ*_sat.F_=0.4 and get *J*_max.F_≈1.4*V(*1+*φ*_L_+*φ*_N_+*φ*_F_) μg_C_ per day and *K*≈350 μg_C  _l^−1^ from Figure 2a. This gives *M*_F_≈14.0962  per day, *α*_F_≈0.1611 l per day per μg_C_, A_max.F_≈4.654 × 10^−7^ l per day.

## References

[bib1] Adolf JE, Stoecker DK, Harding LW. (2006). The balance of autotrophy and heterotrophy during mixotrophic growth of *Karlodinium micrum* (Dinophyceae). J Plankton Res 28: 737–751.

[bib2] Andersen KH, Aksnes DL, Berge T, Fiksen Ø, Visser A. (2015). Modeling emergent trophic strategies in plankton. J Plankton Res 37: 862–868.

[bib3] Bachvaroff TR, Adolf JE, Place AR. (2009). Strain variation in *Karlodinium veneficum* (Dinophyceae): toxin profiles, pigments, and growth characteristics. J Phycol 45: 137–153. 27033653 10.1111/j.1529-8817.2008.00629.x

[bib4] Berge T, Hansen PJ, Moestrup Ø. (2008a). Feeding mechanism, prey specificity and growth in light and dark of the plastidic dinoflagellate *Karlodinium armiger*. Aquat Microb Ecol 50: 279–288.

[bib5] Berge T, Hansen PJ, Moestrup Ø. (2008b). Prey size spectrum and bioenergetics of the mixotrophic dinoflagellate *Karlodinium armiger*. Aquat Microb Ecol 50: 289–299.

[bib6] Berge T. (2011). Functional diversity of marine protists: evidence from culture experiments. PhD thesis, Department of Biology. University of Copenhagen, Copenhagen, Denmark, p 113.

[bib7] Berge T, Poulsen LK, Moldrup M, Hansen PJ. (2012). Marine microalgae attack and feed on metazoans. ISME J 6: 1926–1936. 22513533 10.1038/ismej.2012.29 PMC3446796

[bib8] Berge T, Hansen PJ. (2016). Role of the chloroplasts in the predatory dinoflagellate *Karlodinium armiger*. Mar Ecol Prog Ser 549: 41–54.

[bib9] Bruggeman J, Kooijman SALM. (2007). A biodiversity-inspired approach to aquatic ecosystem modeling. Limnol Oceanogr 52: 1533.

[bib10] Bruggeman J. (2009) Succession in Plankton Communities. The Netherlands: Vrije Universiteit Amsterdam:, pp 71–99.

[bib11] Burkholder JM, Glibert PM, Skelton HM. (2008). Mixotrophy, a major mode of nutrition for harmful algal species in eutrophic waters. Harmful Algae 8: 77–93.

[bib12] Calbet A, Bertos M, Fuentes-Grünewald C, Alacid E, Figueroa R, Renom B et al. (2011). Intraspecific variability in *Karlodinium veneficum*: growth rates, mixotrophy, and lipid composition. Harmful Algae 10: 654–667.

[bib13] Cavalier-Smith T. (1982). The origins of plastids. Biol J Linn Soc 17: 289–306.

[bib14] Falkowski PG, Raven J. (2013) Aquatic Photosynthesis. Princeton University Press, Princeton, NJ, USA.

[bib15] Flynn KJ, Mitra A. (2009). Building the 'perfect beast': modelling mixotrophic plankton. J Plankton Res 31: 965–992.

[bib16] Flynn KJ, Stoecker DE, Mitra A, Raven JA, Glibert PM, Hansen PJ et al. (2013). Misuse of the phytoplankton – zooplankton dichotomy: the need to assign organisms as mixotrophs within plankton functional types. J Plankton Res 35: 5–11.

[bib17] Hansen PJ. (2011). The role of photosynthesis and food uptake for the growth of marine mixotrophic dinoflagellates. J Eukaryot Microbiol 58: 203–214. 21435078 10.1111/j.1550-7408.2011.00537.x

[bib18] Hansen PJ, Nielsen LT, Johnson M, Berge T, Flynn KJ. (2013). Acquired phototrophy in *Mesodinium* and *Dinophysis*–a review of cellular organization, prey selectivity, nutrient uptake and bioenergetics. Harmful Algae 28: 126–139.

[bib19] Hartmann M, Grob C, Tarran GA, Martin AP, Burkill PH, Scanlan DJ et al. (2012). Mixotrophic basis of Atlantic oligotrophic ecosystems. Proc Nat Acad Sci USA 109: 5756–5760. 22451938 10.1073/pnas.1118179109 PMC3326507

[bib20] Irigoien X, Flynn KJ, Harris RP. (2005). Phytoplankton blooms: a ‘loophole'in microzooplankton grazing impact? J Plankton Res 27: 313–321.

[bib21] Jones RI. (1994). Mixotrophy in planktonic protists as a spectrum of nutritional strategies. Mar Microb Food Webs 8: 87–96.

[bib22] Kiørboe T. (2011). How zooplankton feed: mechanisms, traits and trade-offs. Biol Rev Camb Philos Soc 86: 311–339. 20682007 10.1111/j.1469-185X.2010.00148.x

[bib23] Klausmeier CA, Litchman E, Daufresne T, Levin SA. (2004). Optimal nitrogen-to-phosphorus stoichiometry of phytoplankton. Nature 429: 171–174. 15141209 10.1038/nature02454

[bib24] Li AS, Stoecker DK, Adolf JE. (1999). Feeding, pigmentation, photosynthesis and growth of the mixotrophic dinoflagellate *Gyrodinium galatheanum*. Aquat Microb Ecol 19: 163–176.

[bib25] Li A, Stoecker DK, Coats DW. (2000). Mixotrophy in *Gyrodinium galatheanum* (Dinophyceae): grazing responses to light intensity and inorganic nutrients. J Phycol 36: 33–45.

[bib26] Litchman E, Klausmeier CA, Schofield OM, Falkowski PG. (2007). The role of functional traits and trade-offs in structuring phytoplankton communities: scaling from cellular to ecosystem level. Ecol Lett 10: 1170–1181. 17927770 10.1111/j.1461-0248.2007.01117.x

[bib27] Mitra A, Flynn KJ, Burkholder JM, Berge T, Calbet A, Raven JA et al. (2014). The role of mixotrophic protists in the biological carbon pump. Biogeosciences 11: 995–1005.

[bib28] Mitra A, Flynn KJ, Tillmann U, Raven JA, Caron D, Stoecker DK et al. (2016). Defining planktonic protist functional groups on mechanisms for energy and nutrient acquisition: incorporation of diverse mixotrophic strategies. Protist 167: 106–120. 26927496 10.1016/j.protis.2016.01.003

[bib29] Morel A, Bricaud A. (1981). Theoretical results concerning light absorption in a discrete medium, and application to specific absorption of phytoplankton. Deep Sea Res 28: 1375–1981.

[bib30] Norberg J, Swaney DP, Dushoff J, Lin J, Casagrandi R, Levin SA. (2001). Phenotypic diversity and ecosystem functioning in changing environments: a theoretical framework. Proc Natl Acad Sci USA 98: 11376–11381. 11535803 10.1073/pnas.171315998 PMC58737

[bib31] Paasche E, Bryceson I, Tangen K. (1984). Interspecific variation in dark nitrogen uptake by dinoflagellates. J Phycol 20: 394–401.

[bib32] Putt M. (1990). Metabolism of photosynthate in the chloroplast retaining ciliate *Luboea strobila*. Mar Ecol Prog Ser 60: 271–282.

[bib33] Raven J. (1984). A cost-benefit analysis of photon absorption by photosynthetic unicells. New Phytol 98: 593–625.

[bib34] Raven J. (1997). Phagotrophy in phototrophs. Limnol Oceanogr 42: 198–205.

[bib35] Rhee GY, Gotham IJ. (1980). Optimum N:P ratios and coexistence of planktonic algae. J Phycol 16: 468–489.

[bib36] Sheng J, Malkiel E, Katz J, Adolf JE, Place AR. (2010). A dinoflagellate exploits toxins to immobilize prey prior to ingestion. Proc Natl Acad Sci USA 107: 2082–2087. 20133853 10.1073/pnas.0912254107 PMC2836682

[bib37] Skovgaard A. (1996). Mixotrophy in *Fragilidium subglobosum* (Dinophyceae): growth and grazing responses as functions of light intensity. Mar Ecolol Prog Ser 143: 247–253.

[bib38] Skovgaard A, Hansen PJ, Stoecker DK. (2000). Physiology of the mixotrophic dinoflagellate *Fragilidium subglobosum*. I. Effects of phagotrophy and irradiance on photosynthesis and carbon content. Mar Ecol Prog Ser 201: 129–136.

[bib39] Stickney HL, Hood RR, Stoecker DK. (2000). The impact of mixotrophy on planktonic marine ecosystems. Ecol Model 125: 203–230.

[bib40] Stoecker DA. (1998). Conceptual models of mixotrophy in planktonic protists and some ecological and evolutionary implications. Eur J Protistol 34: 281–290.

[bib41] Straile D. (1997). Gross growth efficiencies of protozoan and metazoan zooplankton and their dependence on food concentration, predator–prey weight ratio, and taxonomic group. Limnol Oceanogr 42: 1375–1385.

[bib42] Thingstad TF, Havskum H, Garde K, Riemann B. (1996). On the strategy of 'Eating your competitor': a mathematical analysis of algal mixotrophy. Ecology 77: 2108–2118.

[bib43] Våge S, Castellani M, Giske J, Thingstad TF. (2013). Successful strategies in size structured mixotrophic food webs. Aquat Ecol 47: 329–347.

[bib44] Ward BA, Dutkiewicz S, Barton AD, Follows MJ. (2011). Biophysical aspects of resource acquisition and competition in algal mixotrophs. Am Nat 178: 98–112. 21670581 10.1086/660284

[bib45] Wilken S, Schuurmans M, Matthijs HCP. (2014). Do mixotrophs grow as photoheterotrophs? Photophysiological acclimation of the chrysophyte *Ochromonas danica* after feeding. New Phytol 2044: 882–889. 10.1111/nph.12975 25138174

